# HIV Drug-resistant Strains as Epidemiologic Sentinels

**DOI:** 10.3201/eid1202.050321

**Published:** 2006-02

**Authors:** María S. Sánchez, Robert M. Grant, Travis C. Porco, Wayne M. Getz

**Affiliations:** * University of California, Berkeley, California, USA;; † Gladstone Institute of Virology and Immunology, San Francisco, California, USA;; ‡ California Department of Health Services, Berkeley, California, USA

**Keywords:** acute infection, drug resistance, epidemiology, HIV, infectious disease, mathematical model, population dynamics, prevalence, primary infection, transmission

## Abstract

Observed declines in drug resistance to nucleoside reverse transcriptase inhibitors among persons recently infected with HIV-1 in monitored subpopulations can be interpreted as a positive sign and lead public health officials to decrease efforts towards HIV prevention. By means of a mathematical model, we identified 3 processes that can account for the observed decline: increase in high-risk behavior, decrease in proportion of acutely infected persons whose conditions are treated, and change in treatment efficacy. These processes, singly or in combination, can lead to increases or decreases in disease and drug-resistance prevalence in the general population. We discuss the most appropriate public health response under each scenario and emphasize how further data collection and analyses are required to more reliably evaluate the observed time trends and the relative importance of forces shaping the epidemic. Our study highlights how drug resistance markers can be used as epidemiologic sentinels to devise public health solutions.

## Abstract 2

The decrease in the proportion of drug resistance among newly infected HIV-1 patients may signal a worsening of the epidemic.

In recent years investigators have begun monitoring the HIV epidemic by reporting changes in the proportion of newly infected persons who are carrying an HIV-1 drug-resistant strain, i.e., the primary or acute resistant fraction. Several studies report decreases in this primary resistant fraction (*1**–**7*), including transient decreases (*8**–**14*). Unfortunately, none of these studies included precise longitudinal data on the exact number and type of infected persons or of the fraction of the total population that is screened for acute infection or resistance, and as we show here, making direct interpretations from data collected from a subset of the population can lead to erroneous conclusions. Given the potentially serious clinical implications of drug resistance for HIV-infected persons, public health officials and other authorities need to know whether the decline in drug resistance among acutely infected persons in the monitored subpopulations corresponds to a real decline in drug resistance in the general population and whether this effect is sustainable over time. The decline might be interpreted as a positive sign caused, for example, by less high-risk activity by HIV-positive persons infected with a drug-resistant variant. This explanation could lead public health officials to decrease their support for HIV surveillance and prevention programs targeted at impeding the spread of drug-resistant HIV strains such as drug-resistance testing or adherence counseling. The amount of resources that should be dedicated to drug resistance monitoring and reporting is a controversial issue in light of the recent isolation of a highly virulent multidrug-resistant strain in New York City (*15*). Here we show how drug-resistance data can offer not only clinical information regarding appropriate treatment regimens for individual patients, but also critical insights into an epidemic's course.

## Treatment History of HIV-1 and Its Impact on High-Risk Behavior

Different types of drugs have been developed to fight HIV. Zidovudine (AZT), a nucleoside reverse transcriptase inhibitor (NRTI), was first administered in 1987, and until 1995, monotherapy or dual therapy with NRTIs were the only treatments available. The first protease inhibitor (PI), saquinavir, was approved for treatment in 1995, followed closely in 1996 by a nonnucleoside reverse transcriptase inhibitor (NNRTI), nevirapine. These new drugs generated a major change in the treatment strategy against HIV—highly active antiretroviral therapy (HAART)—that coincided with the start of the monitoring periods in several of the studies mentioned above (1995–1996). With HAART, at least 3 drugs are administered at the same time, which substantially reduces viral load and, compared to results of earlier regimens, increases the life expectancy of patients. These advantages follow because the mutations necessary to confer resistance to HAART are generated at a slower rate and are lost more rapidly than those conferring resistance to monotherapy or dual therapy. Moreover, viral strains resistant to HAART are not as efficient at completing their own life cycle (e.g., their replication rates are lower), they may generate less illness and lower proportion of deaths among infected persons, and the viral strains are less likely to be transmitted to other persons.

The primary resistance time trends observed for NRTIs do not match those observed for the other 2 drug types. For example, in North America some researchers report a decrease in the proportion of persons recently infected with a drug-sensitive HIV strain resistant to NRTIs; this decrease is followed by an increase and subsequent decrease (*11**–**13*). The pattern is particularly noticeable in the study by Grant et al. (*12*), in which NRTI genotypic resistance decreased from ≈30% in 1997 to 5% in 1999, rose to 20% in 2000, and fell to 15% in 2001. Little et al. also found this trend in primary resistance to NNRTIs (*11*). These 2 studies (*11**,**12*) also documented a steady increase in the proportion of persons newly infected with a virus strain resistant to PIs. Here we focused on identifying the likely forces responsible for the time trends exhibited by viral strains resistant to NRTIs. We did so because their time trends are expected to provide better insight into the long-term dynamics of the epidemic than strains resistant to PIs or NNRTIs, given that NRTIs have been administered to more HIV-infected persons and for a longer period than the other 2 types of drugs (*16**,**17*).

Treatment optimism after the initial successes of HAART likely affected the subsequent dynamics of HIV because these favorable treatment outcomes led some persons to increase their high-risk behavior. Later it became apparent that HAART does not completely eliminate HIV from an infected person or impede its transmission. Moreover, when HAART first became implemented, the best strategy was believed to be "hit hard, hit early," because the medical community was trying to limit the expansion of HIV within an infected person's body and ameliorate the gradual deterioration of the patient's immune system. However, HAART can have considerable negative side effects, which affect the functioning of the gastrointestinal system, renal system, pancreas, and liver and produce changes in blood count, allergies, lactic acidosis, and other problems. As a result, treatment began to be delayed to balance the following factors: 1) containing the viral load, 2) minimizing the risk of drug-resistant mutants developing by limiting the amount of treatment time, and 3) reducing negative side effects.

## Modeling Drug Resistance in HIV

Empirical studies have shown that antiretroviral treatment (ARV) produces substantial changes in the viral dynamics at the within-host level that translate into substantial changes at the between-host level (*8**,**18**,**19*). Mathematical and computational models permit us to create simplified versions of complex realities that we can manipulate to further our understanding of their dynamic behavior. Consequently, numerous theoretical studies have investigated the impact of drug therapy on HIV dynamics at both levels (e.g., 20). Initial HIV treatment models (e.g., 21–23) addressed how ARVs might affect the infectiousness of treated persons, and the spread of HIV and its disease-induced deaths. The magnitude of the public health threat created by drug-resistant HIV strains was only recognized later. As a result, Zaric et al. presented a novel model that showed that adhering to treatment regimens would discourage the emergence of multidrug-resistant HIV strains in heterogeneous populations (*24*). Blower et al. developed a relatively simple but revealing deterministic compartmental framework (*25*) that has served as the reference point for most of the modeling studies subsequently done to investigate the effect of ARVs on disease incidence and prevalence, drug-resistance transmission and prevalence, AIDS death rate, and the potential to eradicate the HIV epidemic (*26**–**31*). Dangerfield et al. built a detailed HAART treatment model that accounts for persons in all 4 HIV stages, and the last is partitioned in early- and late-stage AIDS (*32*). They investigated the effects of HAART on HIV incidence and prevalence, assuming different average efficacious periods and assimilation times for HAART, different infectivity probabilities when receiving HAART, and different increases in the mean number of sexual partners.

Blower and Volberding reviewed mathematical studies used to understand the dynamics of a drug-resistant HIV epidemic, predict the incidence and prevalence of drug-resistant HIV strains, evaluate cost-benefit strategies, and assess the impact of public health policies (*33*). The general approach to these studies had been to construct a descriptive simplification of the epidemic by identifying critical categories and processes and to use this structure to make predictions, given a set of assumptions regarding the parameter values. In this regard, several studies have characterized the epidemic's trends as monotonic, including the fraction of new HIV infections that are drug resistant (e.g., 16,25,28,34). Goudsmit et al. conducted an analysis in which including changes in treatment rates explained the nonmonotonic trends of zidovudine resistance observed in a cohort of newly infected homosexual men enrolled in the Amsterdam Cohort Study (*1*).

In our previous study (*35*), we extended the basic modeling framework detailed in (*25*) to incorporate additional complexity, including 2–3 separate categories of acutely infected persons, depending on whether a person was infected with a drug-sensitive HIV strain, a strain resistant to monotherapy, or a strain resistant to triple-drug therapy. In doing so, we were able to distinguish among acutely infected persons, who are clinically and epidemiologically distinct from uninfected and chronically infected persons (e.g., we can consider them to engage more frequently in high-risk behavior (*2*) than chronically infected persons). We also counted these categories separately and tracked their temporal trends and better channeled the different categories through the model, according to the different processes acting on them (such as a decrease in the proportion of persons receiving treatment among those recently infected, an effect that did not occur in persons in the chronic phase). We also created 10 subcategories in the chronically infected stage to more accurately represent the progression of persons from the acute stage of infection to AIDS (*35*). Moreover, because our intent was to explain observed trends rather than to make future predictions, we adopted a specific approach that consisted in altering individually the value of each parameter during a given simulation (as was done in [1] with treatment rates of all persons), rather than running simulations with a set of fixed parameters and comparing outcomes across runs.

## Primary Resistant Fraction

The most direct explanation for the decrease in the observed proportion of newly infected persons infected with NRTI-resistant HIV-1 strains is that resistance to NRTIs in the recently HIV-infected population is declining. Unfortunately, the data need to be further evaluated because the HIV infection status of every person in the general population has not been monitored. We therefore do not have absolute numbers for these time trends, but only the relative numbers obtained from monitored subpopulations, which consist of consenting persons enrolled in research programs at specific locations. To be eligible to participate in these programs, patients had to display symptoms typical of an acute HIV seroconversion syndrome or have recently engaged in risky activities that could have placed them at risk of contracting HIV. Accordingly, what has decreased is the fraction of drug-resistant carriers among the pool of recently infected HIV patients who are willing to participate in particular research programs and attend clinics involved in these studies. The time trends exhibited by the variable representing the actual counts of all the newly infected persons who are carriers for a resistant strain in the general population may or may not be a direct match to those of the monitored subpopulations.

These points are best illustrated by considering the fraction of recently infected persons who are carriers of a drug-resistant strain (primary resistant fraction, *F_R_*), defined mathematically as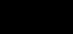,where *S* is the number of persons initially infected with a drug-sensitive strain, and *R* is the number of persons initially infected with a drug-resistant strain. This fraction may decrease because *R* decreases (fewer newly infected persons have a strain that is drug resistant), or because *S* increases (more newly infected persons carry drug-sensitive strains). If both *S* and *R* increase or decrease by the same proportion, *F_R_* remains unchanged. As explained below, the benefit of using this variable's time trends to further our understanding of the past, present, and future of the HIV epidemic is that underlying alterations in the relative values of drug-sensitive and drug-resistant strains may arise from a variety of mechanisms with critically different epidemiologic outcomes.

**Figure 1:**
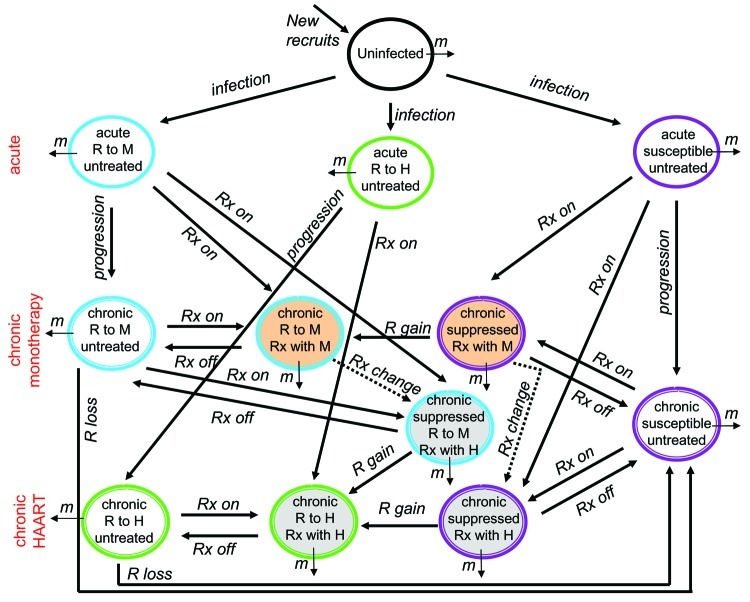
Flow chart of the different categories and flows considered in our model system. For simplicity, we considered 1 type of treatment when analyzing the effects of an increase in high-risk behavior and treatment delay. When considering the effects of overall change in treatment strategy, all categories and flows were included in the analysis. Abbreviations: *m*, mortality (composed of background deaths for all categories, and for persons in the chronic phase of infection, HIV-related deaths are included); Rx, treatment; R, resistance; M, monotherapy; H, highly active antiretroviral therapy (HAART). Color code for the categories' encircling ovals: black (uninfected); purple (wild type); blue (monotherapy resistant strain); green (HAART-resistant strain). Background code of oval categories: no fill (untreated); tan (monotherapy); gray (HAART). Code for the categories' encircling ovals: single, no staging (uninfected and acutely infected); double, staged categories (persons in the chronic phase).

To determine which processes could have caused the observed decrease in F_R_, we built a mathematical model of HIV transmission (Figure 1); a more mathematically detailed explanation of our analysis can be found in our previous study (*35*). We then simulated the epidemic using this model and varied each of the parameters shown in Figure 1 (e.g., the average number of high-risk contacts in 1 year, the likelihood of transmitting HIV given a high-risk contact, the fraction of persons with acute or chronic HIV infection that are placed on treatment each year, the likelihood of generating or losing drug resistance in 1 year). Once we determined which processes can cause a decrease in the acute fraction infected with a drug-resistant strain, we evaluated whether the process had occurred in industrialized countries in recent years. If so, we could consider the process as a potential contributor to the observed trends. As a result, we identified 3 independent processes that caused a decrease in *F_R_* and were consistent with the history of the HIV-1 epidemic in industrialized countries from 1995 to 2001: 1) overall increase in risky behavior, 2) decrease in the fraction of individuals in the acute phase who are placed on treatment, and 3) increase in the efficacy of treatment. Goudsmit et al. also found that discontinuation of monotherapy with zidovudine in 1996 explained the observed drop in zidovudine resistance in patients newly infected with HIV in the Amsterdam Cohort Study (*1*).

**Figure 2:**
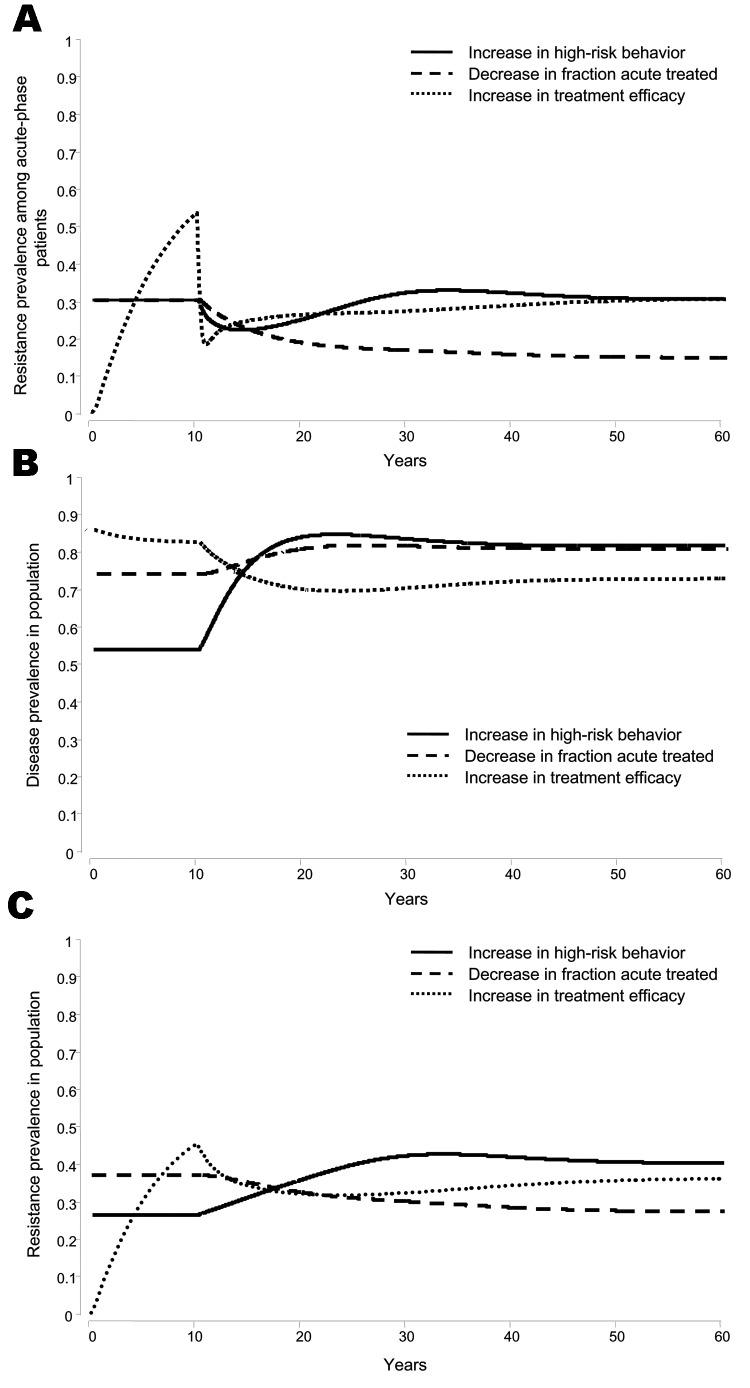
Time trends for A) proportion of persons in the acute phase infected with a resistant viral strain, B) disease prevalence in the population, and C) resistance prevalence in the population. At time *t* = 10 years we introduce a 1) increase in high-risk behavior from 2 to 4 contacts/person/year, or 2) decrease in the yearly fraction of acutely infected persons on treatment from 0.4 to 0.1, or 3) increase in treatment efficacy from monotherapy with zidovudine (AZT) to highly active antiretroviral therapy (HAART). All other parameter values and conditions are as reported by Sánchez et al. (*35*). At *t* = 0, there is 1 infected person in a population of 100,000. For the first 2 processes, we let the simulations reach equilibrium and then introduced the change. The graphs show the trajectories starting at equilibrium and the changes occurring after 10 years. The third process reconstructs San Francisco's historical time frame for the treatment regimen change. Now the epidemic runs without treatment for 30 years, monotherapy with AZT follows for 10 years, and HAART begins at *t* = 40. To facilitate the comparison with the first 2 processes, we graphed the dynamics of the treatment change from the moment AZT was introduced.

Figure 2A illustrates the outcomes of running the model given our manipulation of the parameter values characterizing these 3 processes. We obtained the same qualitative patterns across all reasonable combinations of parameter values. When high-risk behavior increases, the drug-sensitive strain has an initial advantage over the drug-resistant strain because of its higher transmission rate, and it increases to its equilibrium prevalence value at a faster pace than the drug-resistant strain. This increase causes a temporary decrease in *F_R_* (solid trajectory, Figure 2A). The decrease is only temporary because the relative equilibrium prevalence value of the strains is independent of the risky behavior rate, and the relative prevalence value among acute *F_R_* returns to its original value before the perturbation (*35*). If fewer persons are treated, fewer patients will be generating and transmitting drug-resistant strains (dashed trajectory, Figure 2A). The change in treatment efficacy also leads to a decrease in *F_R_* because drug-resistant strains are harder to generate and are less likely to be transmitted under treatment with HAART than under monotherapy with AZT (dotted trajectory, Figure 2A). Figure 2A shows that the long-term behavior of the primary resistant fraction is substantially different under the 3 scenarios, even though it initially decreases for all 3.

## Course of the HIV Epidemic

How do these 3 processes (increase in high-risk behavior, treatment delay, and greater treatment efficacy) impact the course of the HIV epidemic? Are these effects comparable or are they sufficiently different such that the policy implications will vary according to which one we interpret to be the leading cause for the observed decline? To address these questions, we determined the short- and long-term changes induced by these processes on 2 variables of critical public health importance for which we do not have reliable measurements: prevalence of disease (i.e., overall fraction of persons infected with HIV in the general population) and prevalence of drug resistance (i.e., overall fraction of persons infected with a drug-resistant HIV strain in the general population). By doing so, we use time trend changes in the relative prevalence values of 2 viral strains (i.e., the fraction *F_R_* defined above) to make inferences about changes in the absolute values of disease and resistance prevalence in the population (i.e., actual counts of infected persons and carriers of drug-resistant infections in the general population). Figures 2B and 2C show that the 3 factors we identified as causing a decrease in the primary resistant fraction are predicted to force different and permanent, long-term changes in disease and drug-resistance prevalence. A synopsis of our findings is provided in the Table.

**Table:** Impact of an increase in high-risk behavior, decrease in the fraction of acute HIV infected persons receiving treatment, and increase in treatment efficacy on the primary resistant fraction, disease, and resistance prevalence in the overall population

Cause	Effect		
	Primary resistant fraction	Disease prevalence	Resistance prevalence
Increase in high-risk behavior	Temporary decrease	Permanent increase	Permanent increase
Decrease in fraction acute treated	Permanent decrease	Permanent increase	Permanent decrease
Increase in treatment efficacy	Permanent decrease	Permanent decrease	Permanent decrease

## Policy Implications and Comparison with the Data

Our results demonstrate how a decrease in the fraction of persons recently infected with a drug-resistant HIV strain can occur not only when the epidemiologic conditions improve (i.e., disease and drug-resistance prevalence in the population decrease), but also when the epidemic worsens (i.e., disease and drug-resistance prevalence increase). The 3 processes that can generate the decrease in primary resistance are not mutually exclusive, and probably all have contributed to the observed time course of *F_R_*. The challenge now is to identify which one has had the greatest effect on the recent trends of disease and drug-resistance prevalence in the HIV-1 epidemic.

If an increase in high-risk behavior has dominated HIV-1 epidemiology since the onset of HAART, then the decrease in primary resistance, counter to intuition, signals a worsening of the epidemic: a greater number of persons may have become infected, and a greater number of persons may be infected with a viral strain resistant to drug therapy. Other studies have obtained similar conclusions (e.g., 28,29,31,32). If this is the case, the public health response to the decrease in drug-resistance levels among the acutely infected should be to expand programs aimed at reducing high-risk behavior.

Determining the most appropriate public health response is difficult if a decrease in the fraction of acutely ill persons receiving treatment is the main driving force of the HIV epidemic. Under this scenario, the indications for treating acutely infected persons may need to be modified by taking into account the potential balance between an increased number of infected persons as opposed to a decreased number of carriers of drug-resistant infections. Cost-benefit analyses of this nature are an intrinsic part of public health policy (*33*). In any case, we do not expect this process to be the main driving force responsible for recent trends in disease and drug resistance in the HIV-1 epidemic because the number of persons in the acute phase of HIV infection is much smaller than that in the chronic phase (*1*), and a large proportion of chronically infected patients received HAART at the beginning of the study period (*36*). Moreover, new treatment regimens, such as structured treatment interruptions and drug holidays, may have affected recent drug-resistance trends (*36*).

The most favorable outcome occurs if the increase in treatment efficacy brought about with HAART is the most important process determining recent HIV-1 trends. Now both the prevalence of the disease and of drug resistance in the population are decreasing, and therefore the decline in drug-resistance prevalence among the acutely infected is a positive sign (*25**,**26**,**32*). Results under this scenario underscore the importance of public health interventions directed toward increasing the number of persons receiving treatment (we must keep in mind that these results assume that the fraction of persons treated remains constant).

However, the uncertainty in the parameters does not allow us to readily distinguish between the 3 likely scenarios. Moreover, we cannot be sure that the trends observed correspond to a real decrease in *F_R_*, or are simply fluctuations due to stochastic or sampling phenomena around a monotonically increasing time trend (*11**,**18**,**25**,**34**,**37**–**39*). Little et al. and Grant et al. report trends consistent with the first scenario: the decrease in the fraction of persons infected with an NRTI-resistant strain is followed by an increase and subsequent decrease (*11**–**13*). Several authors report biphasic patterns of alternating trends (*1**,**2**,**5**,**9**,**10**,**14*), which are not necessarily correlated with an increase in non-B subtypes (*8*). Other studies report overall increasing (*38*), stable (*40*), and decreasing (*3**,**4**,**6**,**7*) trends in the proportion of persons recently infected with an NRTI drug-resistant HIV-1 strain. These studies, together with our results, highlight why disease surveillance must be increased, with additional data collection and analyses, to fully understand the present and future course of the HIV epidemic. In this regard, mathematical modeling can provide a crucial tool for the correct interpretation of epidemiologic data by identifying the processes responsible for generating observed time trends and characterizing their potential implications for public health programs.

## Conclusion

Our mathematical analysis shows that the observed time trends of measurable quantities from particular subgroups of infected persons (such as primary drug resistance in monitored subpopulations) can correspond to different and unexpected time trends of variables of critical public health interest that are not measured directly in the general population. On the other hand, with the appropriate analyses, information on drug resistant strains can be used not only to guide treatment in individual patients, but also as epidemiologic sentinels to help devise public health solutions. Because changes in the relative value of 2 strains that vary in any of their life history traits (such as their ability to be transmitted, to be suppressed when in the presence of drug therapies, or to lose mutations that confer drug resistance) can show information on an epidemic's trends, the reasoning and methods we used in this study can be applied equally well to understand the epidemiology of any genetically variable microbe.
